# Live imaging of *Drosophila *gonad formation reveals roles for Six4 in regulating germline and somatic cell migration

**DOI:** 10.1186/1471-213X-7-52

**Published:** 2007-05-21

**Authors:** Ivan BN Clark, Andrew P Jarman, David J Finnegan

**Affiliations:** 1 Centres for Integrative Physiology and Neuroscience Research, University of Edinburgh, George Square, Edinburgh EH8 9XD, UK; 2 Institute of Cell Biology, University of Edinburgh, Kings Buildings, Mayfield Road, Edinburgh EH9 3JR, UK

## Abstract

**Background:**

Movement of cells, either as amoeboid individuals or in organised groups, is a key feature of organ formation. Both modes of migration occur during *Drosophila *embryonic gonad development, which therefore provides a paradigm for understanding the contribution of these processes to organ morphogenesis. Gonads of Drosophila are formed from three distinct cell types: primordial germ cells (PGCs), somatic gonadal precursors (SGPs), and in males, male-specific somatic gonadal precursors (msSGPs). These originate in distinct locations and migrate to associate in two intermingled clusters which then compact to form the spherical primitive gonads. PGC movements are well studied, but much less is known of the migratory events and other interactions undergone by their somatic partners. These appear to move in organised groups like, for example, lateral line cells in zebra fish or *Drosophila *ovarian border cells.

**Results:**

We have used time-lapse fluorescence imaging to characterise gonadal cell behaviour in wild type and mutant embryos. We show that the homeodomain transcription factor Six4 is required for the migration of the PGCs and the msSGPs towards the SGPs. We have identified a likely cause of this in the case of PGCs as we have found that Six4 is required for expression of *Hmgcr *which codes for HMGCoA reductase and is necessary for attraction of PGCs by SGPs. Six4 affects msSGP migration by a different pathway as these move normally in *Hmgcr *mutant embryos. Additionally, embryos lacking fully functional Six4 show a novel phenotype in which the SGPs, which originate in distinct clusters, fail to coalesce to form unified gonads.

**Conclusion:**

Our work establishes the *Drosophila *gonad as a model system for the analysis of coordinated cell migrations and morphogenesis using live imaging and demonstrates that Six4 is a key regulator of somatic cell function during gonadogenesis. Our data suggest that the initial association of SGP clusters is under distinct control from the movements that drive gonad compaction.

## Background

The development of the complex organs of metazoans often involves extensive cell migrations. Examples include the development of the lateral line in fish, the formation of the hypaxial limb muscles in vertebrates and the long range migrations of primordial germ cells (PGCs) during gonad formation in many species [1-3]. Studies of cultured cells and unicellular organisms have contributed a large body of knowledge regarding the mechanisms underlying cellular motility [4], and there is increasing interest in understanding migratory events occurring *in vivo *[5,6]. This introduces additional questions relating, for example, to the adhesions between migrating cells and diverse *in vivo *substrates, signalling events governing the direction of migration and the developmental regulation of motile behaviour. While some cell types undergo amoeboid migration in response to cues that are interpreted cell-autonomously, there are many examples of organ morphogenesis involving co-ordinated migration of cells organised in clusters, chains or sheets. The formation of the *Drosophila *embryonic gonads involves several cell types undergoing different modes of migratory behaviour and therefore provides a useful paradigm for studying the cellular interactions leading to organ morphogenesis.

The *Drosophila *PGCs are specified at the posterior pole of the embryo [7] and are carried into the gut cavity during gastrulation, before migrating actively through the midgut epithelium [8,9]. The cells diverge bilaterally away from the midline as they migrate along the basal surface of the midgut and then detach to move to the lateral mesoderm [10]. At around the same time, the somatic gonadal precursors (SGPs) are specified in the dorsolateral mesoderm on either side of the embryo in parasegements 10, 11 and 12 [11,12]. During retraction of the germ band the PGCs are attracted to the SGPs, a process that requires Hmgcr expression by the SGPs, and the two cell types intermingle and compact to form a roughly spherical gonad. A fourth group of somatic cells, the msSGPs, is specified in both sexes in parasegment 13 in a position ventral to the SGPs. The msSGPs migrate as a single cluster to join the posterior of the gonad in males while in females they are present initially but are eliminated by apoptosis during this migration [13,14].

Although factors regulating the migration of the PGCs are well studied, much less is known of the migratory events and other cellular interactions undergone by their somatic partners. A few mutations affect late stages of gonad morphogenesis and all of these are believed to affect the adhesive properties of SGPs [15-19]. Events before this stage are more obscure. After specification, the separate groups of SGPs must come together in the unified gonad, but it is not clear how this occurs.

We have used time-lapse fluorescence microscopy to characterise the behaviour of SGPs and PGCs in living embryos and have found that the transcription factor Six4 regulates several aspects of this behaviour. In embryos containing a hypomorphic allele of *Six4*, SGPs are specified but their function is abnormal as they are unable to attract PGCs, most likely because they fail to express *Hmgcr*. Similarly, the migration of msSGPs to the gonad is impaired. Furthermore the three parasegmental SGP clusters fail to merge to form a unified gonad, indicating a failure in SGP migration or mutual recognition. Our data suggest that the initial association of SGP clusters is under distinct control from the movements that drive gonad compaction.

## Results and discussion

### Imaging cell behaviour during gonadogenesis

The behaviour of PGCs, SGPs and msSGPs was followed by labelling them separately with fluorescent markers: an eGFP-Vasa fusion protein for PGCs [20], and an enhancer from the third intron of *Six4 *[21] to express nls-eGFP in SGPs and msSGPs. Wide field deconvolution microscopy enabled the capture of high resolution, three dimensional image stacks at frequent intervals over an extended period of embryonic development. Wild-type embryos that have been imaged survive to become fertile adults, indicating that our imaging protocols do not significantly perturb gonadogenesis.

For imaging PGC migration, we capture 30 z sections, separated by 1 μm, at 90-second intervals using a 20× objective lens. After mild desiccation of the embryos to reduce their thickness, this is sufficient to observe the path of PGC migration through the mesoderm to the gonads (Additional file 1, Figure 1). Previous observations in fixed tissue suggested that individual PGCs migrate via different paths [22], and our live imaging data confirm this.

**Figure 1:**
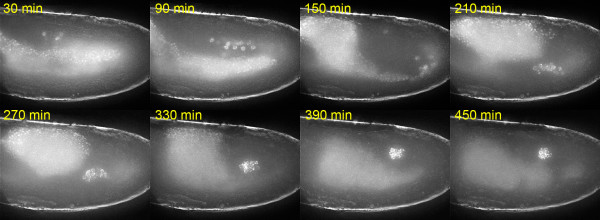
Imaging primordial germ cell movements during gonadogenesis. Embryo expressing eGFP-Vasa in PGCs imaged during stages 11–14. PGCs move to the mesoderm by a variety of routes then coalesce with SGPs (not visible) following germ band retraction. The gonad then compacts to a spherical shape. Projected images from 30 z sections taken at 1 μm intervals are shown at the time points indicated. Image stacks were collected at 90s intervals over 12 hours. These data are also shown in Additional file 1.

To resolve the SGP and msSGP nuclei it is necessary to image at higher magnification and to use longer exposure times to achieve a sufficient range of pixel intensities. We capture 26 sections, separated by 1 μm, at 90s intervals using a 40× objective lens. During germ band retraction in wild-type embryos, the three groups of SGPs coalesce with each other to form a single cluster intermingled with germ cells by the start of stage 13 (Additional file 2, Figure 2a). The movement of the individual SGP clusters relative to each other is difficult to interpret due to the presence of intermingling germ cells. To clarify this we imaged embryos homozygous for a strong allele of *Hmgcr*, in which few PGCs migrate to the mesoderm [23]. In this case it is clear that the three SGP clusters become closely associated by mid stage 12 (Additional file 3, Figure 2b). From stage 13, SGP movements drive the compaction of the gonad. This occurs symmetrically, with SGPs moving both anteriorly and posteriorly towards a central focus (Additional file 2). In *Hmgcr *mutants it is clear that the movements of gonad compaction follow the initial association of the three parasegmental SGP clusters (Additional file 3), implying that these two processes represent distinct cell behaviours at different developmental stages.

**Figure 2:**
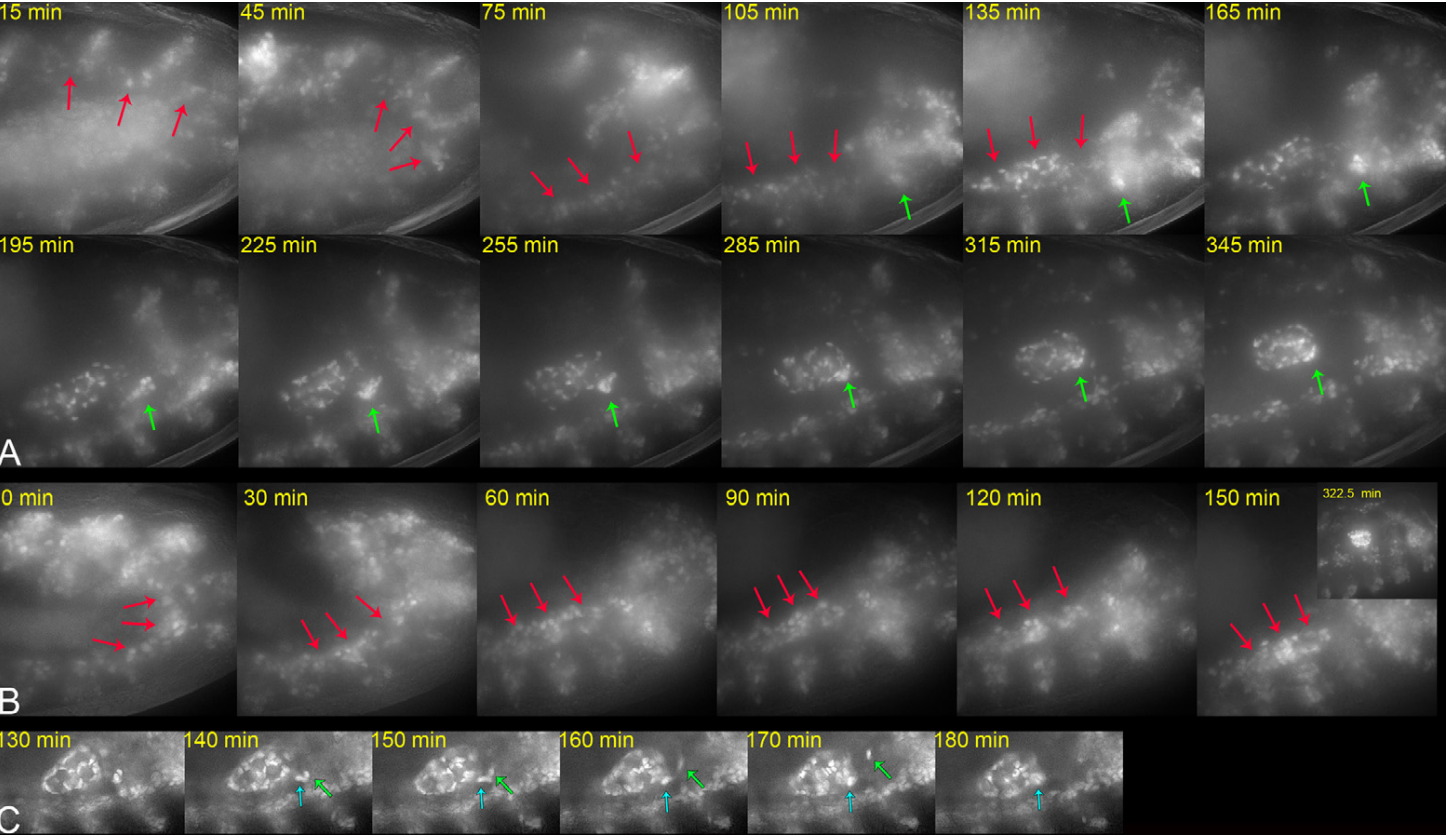
Somatic cell movements during gonadogenesis. **A**. Male embryo carrying a Six4-nls-eGFP transgene imaged during stages 12–14. The three SGP clusters (red arrows) originating in parasegments 10–12 associate during germ band retraction before gonad compaction. The msSGPs (green arrows) migrate anteriorly and dorsally to join the gonad. Projections from 30 z sections taken at 1 μm intervals are shown at the time points indicated. These data are also shown in Additional file 2. **B**. SGP movements in an *Hmgcr *homozygous mutant embryo. Female embryo homozygous for a strong allele of *Hmgcr *allowing SGP movements to be observed in the absence of associating PGCs. The three SGP clusters (red arrows) are in close proximity by mid stage 12 (0 min). These data are also shown in Additional file 3. **C**. Cells migrate towards the gonad from a ventral, posterior position in female embryos. Wild type female embryo expressing nls-eGFP in SGPs. A single section is shown at the time points indicated. A small number of cells migrate to the gonad from parasegment 13 in a similar manner to the msSGPs observed in males. Some remain associated with the gonad (cyan arrow) while others move rapidly away (green arrow). These data are also shown in Additional file 4.

The msSGPs are seen clearly in males as they express nls-GFP to a high level. They migrate anteriorly and dorsally as a tight cluster to join the coalescing gonad at a mean rate of 12.3 microns/hr. This is slower than the rates observed for many cells that migrate individually, but is comparable to the migration rate of border cells during oogenesis, which also move as cohesive cluster [3]. In females, a small number of strongly fluorescing cells migrate from parasegment 13 in a similar manner at this time (Additional file 4, Figure 2c). Observations of fixed tissue [14] suggest that a larger number of msSGPs migrate at this stage in wild type females than we observe, but that most of these undergo apoptosis during this migration. Our data suggest that most of the msSGPs in females do not express nls-eGFP, implying that the *Six4 *enhancer is not active in these cells. The cells that we do observe may be a subset of msSGPs in which the enhancer is active or a distinct cell type. We favour the latter hypothesis because a proportion of cells from the msSGP cluster in males also detach from the gonad shortly after their migration to it (Additional file 2). This suggests that these cells represent a small, distinct population of unknown function that is present in both sexes.

### Six4 is required for PGCs to associate with SGPs

**Figure 3:**
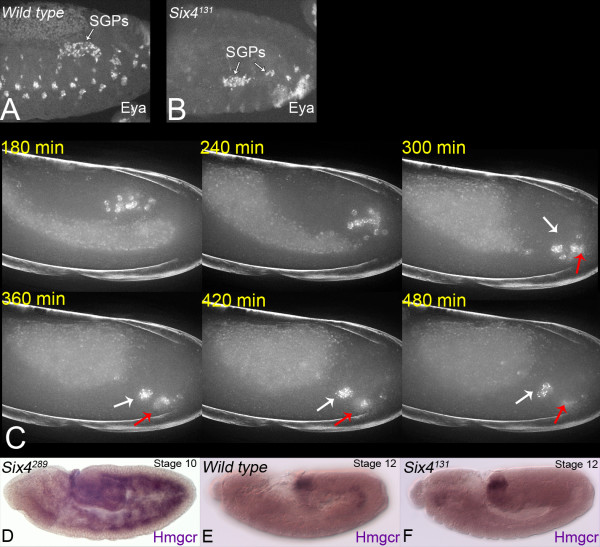
SGPs are present but fail to attract PGCs in *Six4*^*131 *^homozygous embryos. **A**, **B**. Stage 13 wild type (A) and *Six4*^*131 *^homozygous (B) embryos stained for Eya protein. Eya is correctly localised to the nucleus in the mutant SGPs, in contrast to cells lacking Six4 [21, 27]. **C**. Six4 is required for PGC migration to the SGPs. *Six4*^*131 *^homozygous embryo expressing eGFP-Vasa in PGCs. A small number of PGCs (white arrow) become associated with SGPs in the normal location of the gonad following germ band retraction. The majority remain in ventral and posterior positions at this time. Images are shown at the time points indicated and represent maximum projections of data from 30 deconvolved z sections 1 μm apart. These data are also shown in Additional file 5. **D-F**. Six4 is required for *Hmgcr *expression in SGPs. *In situ *hybridisation using an *Hmgcr *RNA probe. D. Stage 10 *Six4 *null embryo. The early, broad expression of *Hmgcr *in the mesoderm is not dependent on Six4. E. By stage 12 *Hmgcr *expression is restricted to the SGPs. F. No *Hmgcr *expression is detected in the SGPs of a *Six4*^*131 *^homozygous embryo.

Although the genetic control of SGP specification is well studied, very little is known about their subsequent development and behaviour. The homeodomain protein Six4 is a candidate regulator of SGP function during gonad formation as it is expressed in the SGPs from their first appearance until at least the end of embryogenesis [24,25]. Although *Six4 *is required for the specification of several mesodermal lineages [21], and for the correct development of the head (unpublished data), embryos homozygous for the hypomorphic mutation *Six4*^*131 *^hatch normally and have only mild musculature defects [24]. These embryos do show a severe defect in gonadogenesis, however. SGPs are present and appear to be correctly specified as they express the markers *412 *[24], Eyes absent (Eya, Figure 3b) and Zfh-1 (not shown) but most PGCs do not associate with them and become scattered.

We have determined the number of SGPs present in embryos stained for Eya and Sox100B expression. At the end of germ band retraction, wild-type male embryos contain 68.5 ± 4.84 Eya-expressing SGPs in each gonadal cluster (n = 4). This number excludes the Sox100B-expressing msSGPs [14]. The number of SGPs is greater than the early estimate of 26–37 that is frequently cited [26]. At a similar stage, male *Six4*^*131 *^homozygotes have 49.6 ± 2.36 (n = 5) Eya-expressing SGPs in this location indicating that a substantial number of SGPs is specified in the *Six4*^*131 *^hypomorph.

The failure of PGC migration in *Six4*^*131 *^embryos is unlikely to be explained by the small reduction in the number of SGPs in this mutant. An alternative explanation is that these SGPs cannot attract the PGCs. Time lapse imaging revealed that PGC migration in *Six4*^*131*^homozygotes begins normally. The cells initially move correctly through the gut epithelium and migrate to the mesoderm but most fail to coalesce into the gonads following germ band retraction (Additional file 5, Figure 3c). The mutant phenotype becomes overt in late stage 12 when most PGCs in wild type embryos undergo a dorsal migration to the final location of the gonad. In *Six4*^*131 *^mutants most PGCs remain in a more ventral and posterior position before dispersing, apparently randomly. This suggests that PGCs move correctly to the mesoderm, but subsequently fail to navigate to the SGPs. Both of these migrations require a chemo-attractant that depends on *Hmgcr *function, firstly in the mesoderm and then the SGPs [23]. In wild type embryos, *Hmgcr *is expressed broadly in the mesoderm at the time that the PGCs detach from the midgut but is then restricted to the SGPs during gonadogenesis. In *Six4*^*131 *^mutant embryos the broad mesodermal expression of *Hmgcr *is still seen but there is no expression in SGPs at the time that they would normally attract the PGCs (Figure 3f). This may explain why PGCs detach correctly from the midgut but many do not enter the gonads in *Six4*^*131 *^mutant embryos. It seems likely therefore that *Hmgcr *is one of the target genes that must be regulated by Six4 for normal SGP function.

### Six4 is required for SGPs to form a unified gonad

Although SGPs do associate in clusters in *Six4*^*131 *^homozygotes [24], time-lapse imaging reveals that there are abnormalities in SGP movement. This indicates that regulation of *Hmgcr *is unlikely to be the only function of Six4 in the SGPs, since gonad formation takes place in *Hmgcr *mutant embryos. In *Six4*^*131 *^mutant embryos the three SGP clusters do not merge to form a single gonadal structure. Of ten mutant embryos that we imaged only one formed a single gonad that incorporated the three parasegmental groups of SGPs. In four embryos the anterior (ps10) cluster remained isolated from the others, while in another four the posterior (ps 12) cluster remained isolated (Additional file 6, Figure 4). In one embryo all three of the SGP clusters remained isolated.

**Figure 4:**
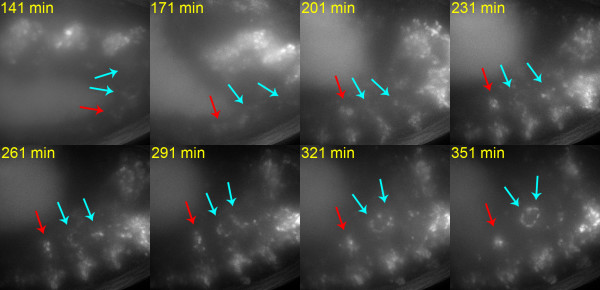
Six4 is required for SGPs to form a unified gonad. *Six4*^*131 *^homozygous embryo carrying a Six4-nls-eGFP transgene. The SGP clusters derived from parasegments 11 and 12 (cyan arrows) associate and compact to form a gonad-like structure in an abnormally posterior position. The cluster derived from parasegment 10 (red arrows) remains isolated but these cells also compact to form a tight cluster. Images shown are at the time points indicated and represent maximum projections of data from 26 deconvolved z sections 1 μm apart. These data are also shown in Additional file 6.

Despite the failure to form a unified gonad, SGPs within these clusters display aspects of their normal behaviour in *Six4*^*131 *^homozygotes. In all of the embryos we examined, a proportion of the SGPs moved towards each other to form a tight cluster, in a process resembling the compaction movements of wild type gonads. In some embryos, isolated clusters of SGPs also compacted so that two gonad-like structures were formed instead of a unified gonad (Additional file 6, Figure 4). Interestingly, several PGCs could be "captured" by these small gonad-like structures, suggesting that short-range PGC-SGP interactions are still possible even though long-range attraction requiring *Hmgcr *is defective. The associating PGCs may be those that randomly move close to the SGPs during their migration. The retention of some SGP behavioural characteristics in *Six4*^*131 *^mutant embryos supports our conclusion that the SGPs are correctly specified. Our interpretation is that most mutant SGPs retain the ability to associate with each other and with PGCs over short distances, but there is a defect in their ability to coalesce over longer distances.

The observation that compaction can occur where SGPs fail to coalesce into a single structure suggests that the two processes differ mechanistically perhaps dependent on distinct short- or long-range interactions respectively the former being Six4-independent. The long range interaction requires Six4 but not E-cadherin as extended clusters of SGPs and PGCs persist in E-Cadherin mutant embryos even though they do not compact into a spherical gonad [15]. As expected, E-Cadherin expression and localisation in SGPs appears normal in *Six4*^*131 *^mutants (data not shown). We suggest that communication between SGP clusters during coalescence requires a Six4-dependent signalling mechanism operating between SGPs. This may direct the migration of the three clusters towards each other prior to compaction, or it may influence the polarity of the compaction process ensuring that a single gonadal cluster is formed. Six4 would positively regulate a component of this signalling pathway, either in the signalling or in the receiving SGPs, or both. When this signalling is disrupted, as in *Six4*^*131*^, compaction may occur via stochastic Six4-independent local cell contacts. This would account for the variability in whether the anterior or posterior SGP cluster fails to be incorporated into the gonad-like structure. Any such signal is unlikely to be *Hmgcr-*dependent, since mutation of *Hmgcr *does not prevent clusters of SGPs from associating, even though they do not attract germ cells (Additional file 3, Figure 2b). An alternative explanation for the SGP coalescence phenotype of *Six4*^*131 *^is that fully functional Six4 is required in SGPs for the compaction process to operate consistently over the distance between SGP clusters, perhaps by regulating the length of productive cellular protrusions that may be required for SGP-SGP contacts.

### Six4 is required for msSGP migration

**Figure 5:**
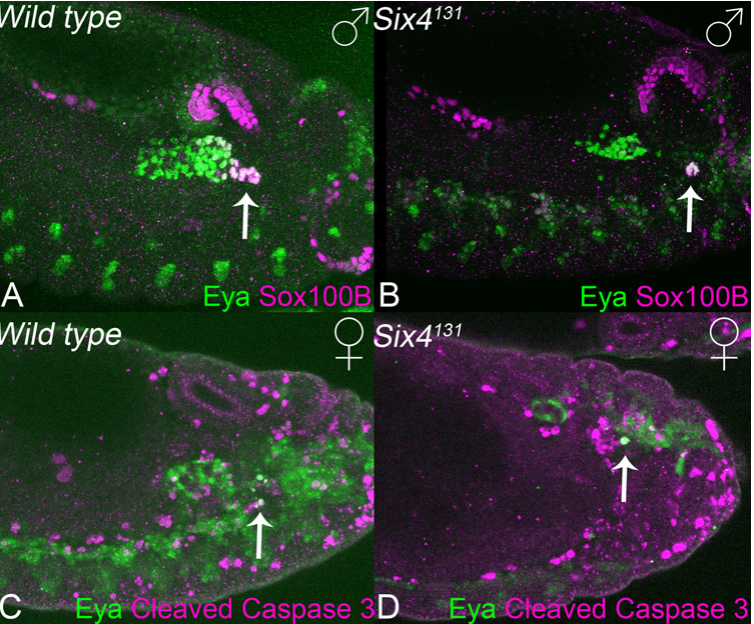
Six4 is required for migration but not specification of msSGPs. Stage 13 male wild type (A) and *Six4*^*131 *^homozygous (B) mutant embryos. The msSGPs (arrows) fail to migrate in the mutant but are maintained as a cluster and express the markers Sox100B and Eya. In females both wild type (C) and *Six4*^*131 *^mutant (D) embryos have cells posterior to the gonads that co-express Eya and an apoptosis marker (arrows), likely to be msSGPs.

The msSGPs must migrate a substantial distance to reach the developing gonad. Given the defects in SGP movements, *Six4*^*131 *^embryos were examined for defects in msSGP migration. In time-lapse experiments, cells originating in parasegment 13 can be identified as msSGPs by the accumulation of high levels of nls-eGFP during stage 13. Unlike wild-type msSGPs, these cells do not migrate but remain in a posterior position (data not shown). Nevertheless, they appear to be correctly specified as they express the msSGP markers Eya and Sox100B [14] and are maintained as a cell cluster in males (Figure 5b) and cells co-expressing both Eya and an apoptosis marker are observed in mutant females as in wild type embryos (Figure 5d). Because Six4 is expressed strongly in msSGPs, as well as SGPs, we cannot yet determine if the failure of msSGPs to migrate may reflect a defect in the msSGPs themselves or defective signalling by the SGPs. The mechanism attracting msSGPs to the gonads is distinct from that attracting the PGCs as they associate correctly with SGPs in *Hmgcr *mutant male embryos (data not shown). It is plausible that msSGP migration and the mutual association of the SGP clusters are regulated by a common mechanism.

A specific role or roles for Six4 in cell migration may not be restricted to the embryonic gonad. A recent study found that Six4 is upregulated in the migratory ovarian border cells with respect to their stationary neighbours, and that Six4 over expression in these cells disrupts their migration [27]. There are similarities in the behaviour of border cells, SGPs and msSGPs, all of which move in cohesive clusters, suggesting that Six4 may influence migratory cell behaviour through regulating some of the same targets at these different stages of reproductive development. Many aspects of Six protein function appear conserved in divergent tissues and organisms [28]. In this context it is interesting to note that cell movements and shape changes during morphogenetic furrow progression in the *Drosophila *eye require the function of *Sine oculis*, another Six gene, while the *C. elegans Six4 *homologue, Unc39, is required both for the specification and the motility of cells migrating anteriorly during embryogenesis [29,30]. The combination of live imaging with the extensive genetic tools available makes the *Drosophila *gonad a productive system to investigate the roles of Six4 transcriptional targets in different modes of cell migration.

## Conclusion

Our work establishes the *Drosophila *gonad as a model system for the analysis of coordinated cell migrations and morphogenesis using live imaging. Using this system we have identified Six4 as a key regulator of cellular movements during this process. In embryos with Six4 function compromised by the hypo-morphic mutation *six4*^*131*^, there are defects in the incorporation of PGCs, SGPs and msSGPs into the gonads. Although the SGPs fail to form a unified gonadal structure, they appear to interact normally with PGCs and with each other over short distances. These observations, and expression of several SGP markers in these cells suggests that they are specified correctly, although we cannot rule out an indirect effect of *six4*^*131 *^on migratory behaviour through a defect in the establishment of cell identity. The mutant phenotype implies that there are separable functions governing the coalescence of gonadal cells and their subsequent morphogenetic movements during gonad compaction and that these functions are under distinct genetic control.

## Methods

### Fly stocks

Wild-type flies were of the Oregon R stock. Flies expressing eGFP-Vasa in the primordial germ cells were provided by Satoru Kobayashi [20]. The *Six4 *alleles and the nls-eGFP reporter driven by the Six4 third intron enhancer were described previously [21,24].

### Time-lapse microscopy

Live embryos were collected, dechorionated, washed and mounted on coverslips in halocarbon oil as described [31]. Images were collected using a DeltaVision wide field fluorescence microscope (Applied Precision) using 20×/0.75NA (PGC Videos) and 40×/1.35NA (SGP/msSGP Videos) objective lenses and a custom GFP filter cube. Up to 4 embryos were imaged in parallel by repeat visiting using a highly accurate XYZ motorised stage. Images were collected using a Roper Coolsnap HQ camera and binned (2 × 2) to increase the range of intensities. Out of focus light was reassigned by iterative deconvolution [31]. Images and Videos were prepared for presentation using Softworx (Applied Precision), Image J (NIH), Photoshop (Adobe) and Quicktime (Apple) software. The mean rate of msSGP migration was calculated from two male embryos from the positions of the centroids of shapes drawn around the msSGP cluster at different time points using Image Pro Plus (Media Cybernetics).

### Histochemistry

Antibody staining was performed on whole mount embryos by standard methods. Detection was by secondary antibodies conjugated to Alexa 488 or 568 fluorochromes (Molecular Probes). The primary antibodies were Eya (mouse 1/50, Developmental Studies Hybridoma Bank, developed by N. Bonnini), Sox100B (gift of Steven Russell), Zfh-1 (1/500 mouse, provided by Z-C Lai), DCAD2 (rat 1/50, Developmental Studies Hybridoma Bank) and cleaved human caspase-3 (Asp715, 1/100, Cell Signalling Technology). For *in situ *hybridisation an *Hmgcr *probe was made by transcription of plasmid pNB36C [23] using Roche reagents. Hybridisation was carried out by standard methods and detected using a secondary antibody conjugated to alkaline phosphatase (Roche). Still images were collected using a Provis (Olympus) microscope or a Pascal (Zeiss) confocal.

## Abbreviations

Eya, Eyes absent; Hmgcr, 3-hydroxy-3-methylglutaryl coenzyme A reductase; msSGP, male-specific somatic gonadal precursor; PGC, primordial germ cell; SGP, somatic gonadal precursor.

## Authors' contributions

IC carried out the experimental work and the analysis and presentation of the data. All of the authors participated in the conception, design and management of the study and the drafting of the manuscript.

## Supplementary Material

Additional file 1 PGC migration to the gonads. An embryo expressing eGFP-Vasa in primordial germ cells imaged during stages 11–14. Germ cells move to the mesoderm by a variety of routes then coalesce with SGPs (not visible) following germ band retraction. The gonad then compacts to a spherical shape. Image stacks were collected at 90s intervals over 12 hours of embryonic development. The video was made from maximum projections of data from 30 deconvolved z sections at each time point taken at 1 μm intervals. The frame rate is 29.97 fps. Still images derived from these data are shown in Figure 1. Click here for file

Additional file 2 Somatic cell movements during gonadogenesis. Male wild type embryo carrying a Six4-nls-eGFP transgene imaged during stages 12–14. The three SGP clusters originating in parasegments 10–12 associate during germ band retraction before further SGP movements result in compaction of the gonad. The msSGPs migrate anteriorly and dorsally to join the gonad. Image stacks were captured at 90s intervals. The video was made from maximum projections of data from 26 deconvolved z sections at each time point, taken at 1 μm intervals. The frame rate is 29.97 fps. Still images derived from these data are shown in Figure 2a Click here for file

Additional file 3 SGP movements in an *Hmgcr *homozygous mutant embryo. Female *Hmgcr *homozygous embryo carrying a Six4-nls-eGFP transgene imaged during stages 12–14. The *Hmgcr *mutation allows SGP movements to be observed in the absence of associating PGCs. The three SGP clusters (red arrows) are in close proximity by mid stage 12 and remain associated for some time before the initiation of gonad compaction. The video was made from maximum projections of data from 26 deconvolved z sections taken at 1 μm intervals. Image stacks were captured at 90s intervals. The frame rate is 29.97 fps. Still images derived from these data are shown in Figure 2b. Click here for file

Additional file 4 Cells migrate towards the gonad from a ventral, posterior position in female embryos. Wild type female embryo carrying a *Six4-nls-eGFP *transgene. A small number of cells migrate to the gonad from parasegment 13 in a similar manner to the msSGPs observed in males. Some remain associated with the gonad while others move rapidly away. Images were captured at 2 minute intervals. The video was made from a single z section at each time point. The frame rate is 29.97 fps. Still images derived from this data are shown in Figure 2c. Click here for file

Additional file 5 Six4 is required for PGC migration to the SGPs. *Six4*^*131 *^homozygous mutant embryo expressing eGFP-Vasa in PGCs. A small number of PGCs (white arrow) become associated with SGPs in the normal location of the gonad following germ band retraction. The majority remain in ventral and posterior positions at this time. The video was made from maximum projections of data from 30 deconvolved z sections 1 μm apart. Image stacks were collected at 90s intervals. The frame rate is 29.97 fps. Still images derived from these data are shown in Figure 3c. Click here for file

Additional file 6 Six4 is required for SGPs to form a unified gonad. *Six4*^*131 *^homozygous mutant embryo carrying a Six4-nls-eGFP transgene. The SGP clusters derived from parasegments 11 and 12 (cyan arrows) associate and compact to form a gonad-like structure in an abnormally posterior position. The cluster derived from parasegment 10 (red arrows) remains isolated but these cells also compact to form a tight cluster. The video was made from maximum projections of data from 26 deconvolved z sections 1 μm apart. The frame rate is 29.97 fps. Image stacks were collected at 90s intervals. Still images derived from these data are shown in Figure 4. Click here for file
